# Supplementary material for: Exploring the Sensitivity of Prodromal Dementia with Lewy Bodies Research Criteria
Source: Brain Sci. 2022 Nov 22;12(12):1594. doi: 10.3390/brainsci12121594 (PMC9775171; doi:10.3390/brainsci12121594)
Supplement: Supplementary file 1 [file brainsci-12-01594-s001.zip › brainsci-2027588-supplementary.pdf]

Supplementary Information:

Table S1. Research Criteria for Prodromal Dementia with Lewy Bodies (as taken from McKeith et al. 2020).

Essential for a diagnosis of prodromal DLB is MCI defined by the presence of each of the following:

Concern by the patient, informant, or clinician regarding cognitive decline.

Objective evidence of impairment in 1 or more cognitive domains. The cognitive impairment may include any domain, but is more likely to be associated with attention-executive and/or visual processing deficits.

Preserved or minimally affected performance of previously attained independence in functional abilities, which do not meet the criteria for dementia.

Core clinical features

Cognition fluctuation with variations in attention and alertness

Recurrent visual hallucinations.

RBD.

One or more spontaneous cardinal features of parkinsonism: these are bradykinesia (defined as slowness of movement and decrement in amplitude or speed), rest tremor, or rigidity.

Proposed biomarkers

Reduced dopamine transporter uptake in basal ganglia demonstrated by SPECT or PET.

Polysomnographic confirmation of REM sleep without atonia.

Reduced meta-iodobenzylguanidine (MIBG) uptake on myocardial scintigraphy

***Probable* MCI-LB can be diagnosed if:**

Two or more core clinical features of DLB are present, with or without the presence of a proposed biomarker, or

Only 1 core clinical feature is present, but with 1 or more proposed biomarkers.

***Probable* MCI-LB should not be diagnosed based on biomarkers alone.**

***Possible* MCI-LB can be diagnosed if:**

Only 1 core clinical feature of DLB is present, with no proposed biomarkers, or

One or more of the proposed biomarkers is present, but there are no core clinical features.

Supportive clinical features

Severe sensitivity to antipsychotic agents; postural instability; repeated falls; syncope or other transient episodes of unresponsiveness; prolonged or recurrent delirium; autonomic dysfunction, e.g., constipation, orthostatic hypotension, urinary incontinence; hypersomnia; hyposmia; hallucinations in other modalities including passage, and sense of presence phenomena; systematized delusions; apathy, anxiety, and depression.

Potential biomarkers of MCI-LB

Quantitative EEG showing slowing and dominant frequency variability.

Relative preservation of medial temporal lobe structures on structural imaging.

Insular thinning and gray matter volume loss on MRI.

Low occipital uptake on perfusion/metabolism scan.

MCI plus supportive clinical features or potential biomarkers are insufficient to diagnose MCI-LB but may raise suspicion of it and prompt biomarker investigation and may add weight to an existing MCI-LB diagnosis.

MCI-LB is less likely in the presence of any other physical illness or brain disease including cerebrovascular disease, sufficient to account in part or in total for the clinical picture, although these do not exclude an MCI-LB diagnosis and may serve to indicate mixed or multiple pathologies contributing to the clinical presentation.

Note: DLB = dementia with Lewy bodies; MCI = mild cognitive impairment; MCI-LB = MCI with Lewy bodies.

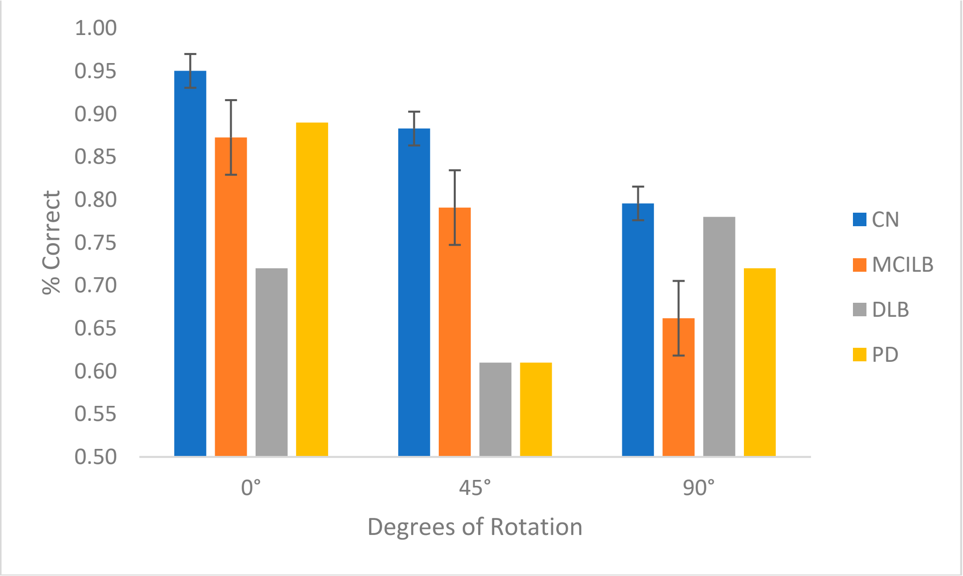

Figure S1. Accuracy for the mental rotation task**.**

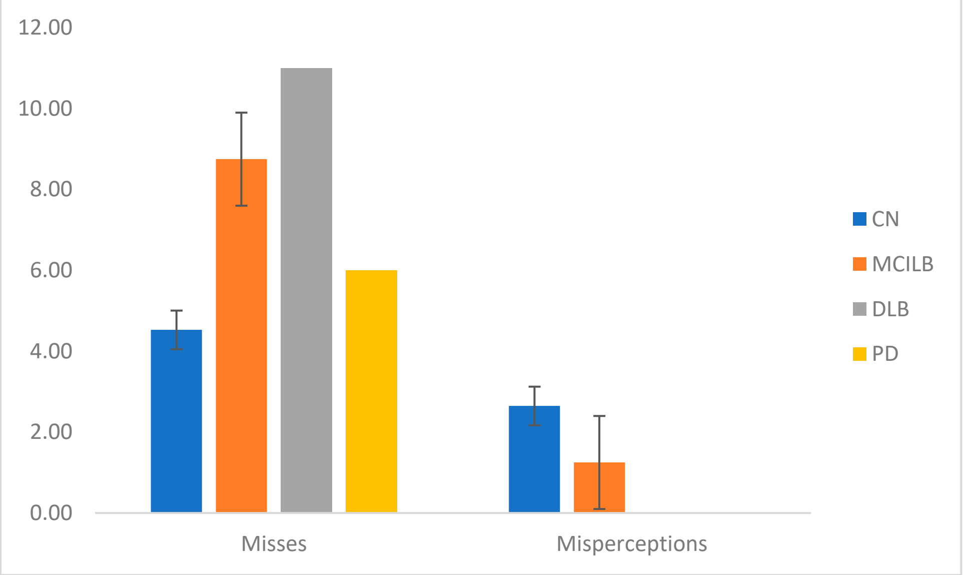

Note: Neither the DLB patient or PD patient made any misperceptions

Figure S2. Performance for Bistable Percept Paradigm.

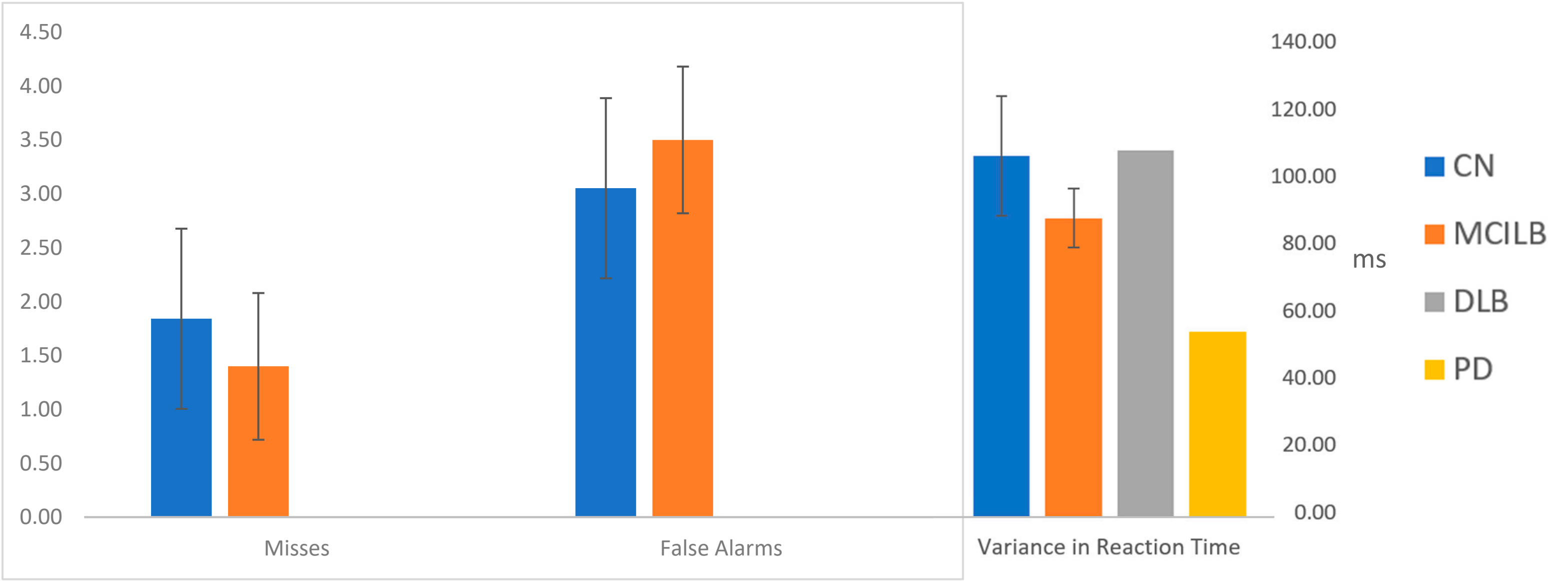

Note: Neither the DLB or PD patients made any misses of false alarms.

Figure S3. Performance for the Sustained Attentional Response Task.
